# Feasibility of using WeChat to improve infant and young child feeding in rural areas in China: A mixed quantitative and qualitative study

**DOI:** 10.1371/journal.pone.0246942

**Published:** 2021-02-25

**Authors:** Qiong Wu, Yiwen Huang, Michelle Helena van Velthoven, Wei Wang, Suying Chang, Yanfeng Zhang

**Affiliations:** 1 Department of Integrated Early Childhood Development, Capital Institute of Pediatrics, Beijing, China; 2 Department of Paediatrics, University of Oxford, Oxford, United Kingdom; 3 Health and Nutrition, Water, Environment and Sanitation Section, UNICEF China, Beijing, China; Helen Keller International, SIERRA LEONE

## Abstract

**Background:**

Appropriate infant and young child feeding practices are the basis for child nutrition. In China, WeChat is gradually changing the channels through which people receive information. The paper aims to explore the feasibility of using WeChat to improve infant and young child feeding in rural China.

**Methods:**

A mixed-methods study was carried out in Huzhu County, Qinghai province, China. We conducted two cross-sectional surveys with children aged 6–23 months and their caregivers in 2012 (N = 1804) and 2018 (N = 754), respectively. Quantitative data were collected on feeding knowledge and practices, caregiver’s use of smartphones and WeChat. Qualitative data were from 33 semi-structured interviews with pregnant women and mothers. In addition, we developed a WeChat feeding health education platform and asked women about their experiences with using it.

**Results:**

In both cross-sectional surveys, less than 10% of caregivers knew that breastfeeding can be continued up to two years, less than 50% knew the accurate duration of exclusive breastfeeding, and only around 20% knew meat can be given to children from the age of 6–8 months. Similarly, the feeding practices were suboptimal and most key infant feeding practices did not change over the years. Only around 30% of caregivers ever received feeding information during pregnancy or after delivery in both surveys. Around 50% of caregivers received information from their relatives and friends, followed by 30% from health facilities and communities. More than 80% of mothers were currently using both a smartphone and the WeChat app, and 75.4% of them were willing to receive feeding information from WeChat official accounts. The WeChat feeding health education platform developed by our study team was generally well accepted by women.

**Conclusions:**

There was an absence of accurate information sources on infant feeding and child nutrition. WeChat could be a potential way to deliver infant feeding recommendations to pregnant women and mothers in rural China.

**Trial registration:**

ChiCTR-PRC-11001446 (The controlled intervention trial for complementary food supplements “Yingyangbao”); ChiCTR1800017364 (the randomized controlled trial for WeChat)

## Introduction

Appropriate nutrition in the first few years of life is extremely important to childhood growth and development, and has lasting effects on children’s future health [1]. The critical window for child nutrition is within the first 1,000 days between conception and the age of two, and any nutritional deficits (such as anemia and stunting) acquired during this period can cause irreversible damage [2, 3]. The promotion of appropriate feeding practices can reduce the incidence of stunting and lead to better health and growth outcomes [4, 5]. Therefore, the World Health Organization (WHO) and United Nations Children’s Fund (UNICEF) recommend exclusive breastfeeding from birth to six months of age followed by the introduction of adequate complementary foods at six months and continued breastfeeding for up to two years [6].

During the past two decades, the Chinese government has made great efforts to improve infant and young child feeding. In 1998, the Ministry of Health introduced the WHO integrated management of childhood illness (IMCI) guidelines and has since implemented IMCI program in rural areas China, in which infant feeding counseling play an important part [7]. Also the Chinese infant and young child feeding (IYCF) strategy was released in 2007 by the Ministry of Health [8]. Many hospitals have been made baby-friendly to promote breastfeeding and breastfeeding week activities were organized to raise public awareness of breastfeeding in China [9–11]. Moreover, a national program called “Basic Public Health Service” has been implemented in China since 2009, in which health care workers are required to provide face-to-face breastfeeding and complementary feeding counseling to pregnant women and mothers throughout antenatal and postnatal care [12]. However, the exclusive breastfeeding rate of babies aged up to 6 months was only 20.7% in China in 2013 and the prevalence of minimum dietary diversity, meal frequency and acceptable diet among children aged 6–23 months was 53.7%, 69.1%, and 25.1%, respectively [13]. In addition, too early or too late introduction of complementary food is very common; around 20% of children younger than 4 months in poor rural areas were given complementary food, and 15% were introduced to complementary food when they were older than 9 months [13].

Women’s knowledge on infant feeding practices is crucial for the health and nutrition wellbeing of a child, which can be improved by healthcare education messages [14–16]. Currently, infant and young child feeding counseling have generally been implemented through the rural three-tier healthcare system (county-township-village) in China [12]. However, previous studies indicated that caregivers in rural areas receive feeding information mainly from family members, friends or their own experience, and rarely from health facilities [17–19]. Thus, child feeding education need to be enhanced and exploring effective ways to promote feeding practices is greatly needed.

With the widespread use of smartphones, interventions delivered through smartphone apps have been increasingly used for health promotion, such as for providing education on antibiotics or diabetes, reducing alcohol consumption, smoking cessation, or to improve diet, physical activity and reduce sedentary behaviour [20–24]. In China, the most popular smartphone app is WeChat (Tencent), which is regarded as one of the leading social networks worldwide. Founded in 2011, WeChat has 902 million daily users, and about 38 billion messages are sent on the platform every day [25]. Furthermore, in addition to messaging, WeChat offers users functions such as video calls, sharing photos and ‘moments’, games, payment, booking flight or hotels. One of the most popular functional modules of WeChat called ‘WeChat official accounts’ can be used for developers, merchants, celebrities, and organizations to communicate and interact with their audience through text, images, voice, videos and rich-media messages [26]. WeChat is gradually changing the ways people receive information, and has been used as a communication tool to change health behaviors, for example for cancer, malaria, asthma, chronic rhinosinusitis, diabetes and weight loss [27–32]. However, no studies have focused on using WeChat to support caregivers with infant and young child feeding. Therefore, this current paper aims to explore the feasibility of using WeChat as a potential way to improve infant and young child feeding in rural China.

## Materials and methods

### Study design and data sources

Mixed methods were used to explore the feasibility of using WeChat as a potential way to improve infant and young child feeding in Huzhu County, Qinghai province, China.

Quantitative data were derived from two cross-sectional surveys, which collected data on caregivers’ infant feeding knowledge and practices, information sources, and use of smartphones and WeChat. From 2012 to 2014, we carried out a controlled intervention trial study to evaluate the effectiveness of complementary food supplements and dietary counseling on nutrition of children aged 6–23 months in Qinghai province [33], and a cross-sectional survey was conducted in 2012 in Huzhu County as the baseline of the trial, which was the first survey for this paper. The intervention was continued to be implemented in Huzhu County after the trial study ended, and we carried out another cross-sectional survey in August 2018 to assess sustainability of intervention, which was the second survey for this paper.

Qualitative data were derived from 33 semi-structured interviews with pregnant women in their second and third trimester and mothers who had a child aged 0–6 months. These semi-structured interviews were conducted in September 2018 to obtain a better understanding of mothers’ child feeding knowledge and the feasibility of using WeChat as an infant feeding education platform.

We integrated quantitative and qualitative data to provide insights into the feasibility of using WeChat as potential way to improve infant and young child feeding. We first report quantitative data followed by qualitative data on infant feeding knowledge and practices, information sources and WeChat use.

### Study setting

This study was conducted in Huzhu County, Qinghai Province, China, which was the intervention county of the controlled intervention trial study [33]. Qinghai province lies in northwest China, with a total population of 5,838,000 in 2017. There are 34 counties and 439 townships in Qinghai Province. Huzhu County, located in the northeast of Qinghai province, has a total population of 401,540 of which the rural population accounts for 76.0%. Huzhu County has 19 townships and 294 villages. The annual per capita income of rural residents in 2017 was ¥9810 (US$1414.91) [34].

### Quantitative study population

Children aged 6–23 months and their caregivers in Huzhu County were participants for the two cross-sectional surveys. Children with a structural or genetic birth defect such as neural tube defects, congenital heart disease or phenylketonuria or caregivers who refused to participate were excluded.

### Quantitative sample size and sampling

For the survey in 2012, the sample size calculation and the sampling was based on the study design of the intervention trial [33]. We expected to achieve a 20% reduction in the prevalence of stunting. With a power of 80% and 5% significance level, we calculated that a sample size of 1793 children aged 6–23 months per group would be sufficient. We used a two-stage sampling procedure to select children. At the first stage, we selected 150 villages out of 294 villages using proportional to population size sampling. At the second stage, we randomly selected and interviewed caregivers of 12 children from the name list of all eligible children in each village [33]. For the survey in 2018, the sample size was calculated based on data from survey in 2012 [33]. We expected to achieve a 20%-point reduction of anemia prevalence and 20–50% points increase for knowledge and appropriate feeding practices. With 80% power and 5% significance level, we estimated that a sample size of 554 children aged 6–23 months would be sufficient for all key indicators. We over-sampled 30% of children to compensate for possible refusal and loss to follow-up. We randomly selected 38 villages from the 150 villages and surveyed all eligible children aged 6–23 months in those villages.

The participants in the two surveys were not the same. In each survey, we randomly selected children aged 6–23 in the selected villages.

### Quantitative data collection

We used the adapted Maternal, Newborn and Child Health household survey (MNCH HHS) tool to collect quantitative data in two cross-sectional surveys, which included socio-demographic characteristics, infant and young child feeding and common illnesses. In addition, we also collected caregivers’ use of smartphones and WeChat during the survey in 2018. We set up all the questionnaires in the specially developed software on smartphones, which interviewers used to record data [35]. Staff from the Capital Institute of Pediatrics in Beijing acted as supervisors in both surveys, and students were recruited from the School of Public Health, Qinghai University in 2012 and Qinghai Institute of Health Sciences in 2018 as interviewers. Two days’ training for each survey was carried out before the fieldwork, including communication skills, explanation of questionnaires, demonstration, role plays, field practice, and group discussions. During each survey, we asked caregivers first to come to register at village clinics, then interviewers introduced the aim of the survey, obtained written informed consent and conducted interviews with caregivers.

### Quantitative data analysis

Data were automatically transferred into a Microsoft Excel sheet. After data cleaning, we converted the database into a database file (dbf) for the analysis. We carried out statistical analysis with SAS 9.2 for Windows. The median (Q1, Q3) was used to describe the age in years of mothers and grandparents. Percentages were presented for binary or categorical variables. We used the Pearson χ^2^–test and Fisher exact test to compare binary or categorical variables.

Five core feeding practice indicators were used to assess feeding practices according to the WHO guidelines ‘Indicators for assessing infant and young child feeding practices (Box 1) [36].

**Box 1. WHO core feeding practice indicators.**

**Introduction of solid, semi-solid or soft foods:** the proportion of infants 6–8 months of age who receive solid, semi-solid or soft foods.

**Minimum dietary diversity:** the indictor was four out of seven food groups per day. The proportion of children aged 6–23 months who receive foods from four or more food groups was estimated. The food groups were: a) grains, root, and tubers; b) legumes and nuts; c) dairy products (milk, yogurt, cheese); d) meat (meat, fish, poultry and liver/organ meat); e) eggs; f) vitamin-A rich fruits and green vegetables; g) other fruits and vegetables.

**Minimum meal frequency:** the proportion of breastfed and non-breastfed children aged 6–23 months who received solid, semi-solid, or soft foods (also including milk for non-breastfed children) the minimum number of times or more.

**Minimum acceptable diet:** Proportion of children aged 6–23 months who reached a minimum dietary diversity and minimum meal frequency.

**Consumption of iron-rich or iron-fortified foods:** Proportion of children aged 6–23 months who received iron-rich food or iron-fortified food that was specially designed for infants and young children, or that was fortified in the home.

The feasibility of using WeChat as a potential way to improve infant and young child feeding was mainly assessed by samrtphone and WeChat usage coverage among caregivers, and caregivers who were willing to receive IYCF information from WeChat official accounts.

### Qualitative semi-structured interview participants and sampling

The participants in the qualitative interviews were independent from the two cross-sectional surveys in the quantitative data collection. We assumed that pregnant and mothers may well accept WeChat to deliver infant feeding recommendations. Therefore pregnant women in their second and third trimester and mothers who had a child aged 0–6 months were the study population for the qualitative semi-structured interviews. Based on our previous data, only around half main caregivers of children aged 6–23 months were mothers, and thus we did not include mothers of children aged 6–23 months in the qualitative semi-structured interviews.

We used convenience sampling to select participants in Huzhu County Maternal and Child Health Family Planning Service Centre. A total of 33 women agreed to participate the qualitative interviews, no one refused. Seventeen pregnant women in second and third trimester who came to the center to receive antenatal care were invited. Seven mothers who just gave birth in the maternity ward of the center and 9 mothers who took their children to receive child health care in the child health care clinic of the center were also invited to be interviewed.

### The WeChat feeding health education platform

Before conducting the qualitative interviews, the study team developed a WeChat feeding health education platform on the local WeChat official account “Huzhu County Maternal and Child Health Family Planning Service Centre” in May 2018, which has describe in detail in our previous paper [37, 38]. We used this platform as an infant feeding intervention in a randomized controlled trial to evaluate the effectiveness on exclusive breastfeeding rate under six months in Huzhu County from May 2019 to April 2020 [37, 38].

The WeChat platform includes a feeding messages, feeding knowledge competition, baby growth chart, and online forum. The feeding messages module is for providing key breastfeeding knowledge, recommendations, and breastfeeding problems encountered for both mothers and children in the form of text, videos, and pictures. All the education messages show in the form of text, videos, and pictures. The feeding knowledge competition component aims to test how well mothers know the breastfeeding knowledge. The baby growth chart component is developed based on the WHO growth chart standard. Mothers can enter data on weight and height of their children whenever they want to monitor their children’s growth. The online forum component is designed to enable interaction between mothers and experts. And detailed description of each component was reported in our previous paper [37, 38].

### Qualitative data collection

One researcher from the Capital Institute of Pediatrics (HYW (MD, graduate student, female)) conducted the face-to-face semi-structured interviews in September 2018. The researcher had qualitative interview experience before and was trained on the qualitative methodology before the interviews again. The study team developed the interview guides to understand women’s views on exclusive breastfeeding, their sources of feeding information, which were pilot tested in Huzhu County in July 2018. In addition, we also asked each pregnant woman and mothers to use the WeChat feeding health education platform and asked them for their experiences on use and satisfaction. After signing the written informed consent, participants were invited for an interview in a separate room in Huzhu County Maternal and Child Health Family Planning Service Centre, and no one else was present beside the participants and the researcher. Interviews were conducted in Mandarin, typically lasting for around 60 minutes, and were digitally recorded with the permission of each participant. Tape recordings were transcribed verbatim in Chinese by five medical students from Qinghai Institute of Health Sciences, and then the study team member who conducted the in-depth interviews validated the transcripts.

### Qualitative data analysis

Content analysis was used to examine the major themes and patterns that emerged from the data. Two Chinese researchers involved in the study (WQ (MD, senior researcher, female, had qualitative interview and data analysis experience) and HYW) first read the transcripts and used MAXQDA 11 to identify themes from the data and code them independently. Then the researchers compared the themes and discussed areas of agreement and discrepancies. They further refined the themes until consensus was reached on the themes and interpretation of the findings. A total of 1053 data coders were coded. We listed all the key themes that we identified. The main coding tree was as follows: the feeding information they wanted to know, the sources of feeding knowledge, exclusive breastfeeding conception and duration, exclusive breastfeeding practice, smartphone usage, access the internet by phones, WeChat usage, WeChat official account usage, WeChat platform usage.

### Ethical considerations

The study was approved by the Ethics Committee of the Capital Institute of Pediatrics. All interviewees read the Information Sheet and provided written consent. For participants who were illiterate or could not write, they asked village doctors to help them to sign their name on the sheets.

## Results

### Quantitative surveys’ results

#### Participants

A total of 1804 caregivers of children were surveyed in 2012 and 754 in 2018. In both surveys, mothers and grandparents were the main caregivers, accounting for around 50% and 45% respectively (Table 1). For the 6–11 months child age group, more than 65% of main caregivers were mothers in both surveys. Mothers who attended junior high school or above increased from 59.8% in 2012to 79.0% in 2018 (P<0.001) and only 4.0% of mothers were illiterate in 2018. The illiteracy rate for grandparents decreased over time but was still very high; 69.1% in 2012 and 60.3% in 2018, and this decrease was not significant (P = 0.057).

**Table 1:** Children’s main caregivers and their education in both surveys.

	Survey in 2012			Survey in 2018			*P-*values^a^
	6–11 months (N = 610)	12–23 months (N = 1194)	Total (N = 1804)	6–11 months (N = 247)	12–23 months (N = 507)	Total (N = 754)	
**Main caregivers**	<0.001						
Mother	65.6	46.9	53.2	66.4	40.0	48.7	
Grandparents	33.9	50.7	45.0	27.5	55.6	46.4	
Father	0.0	0.9	0.6	6.1	4.2	4.8	
Other	0.5	1.5	1.2	0.0	0.2	0.1	
**Mothers**							
Age in years (median (Q1, Q3))	25 (23, 30)	26 (23, 30)	26 (23, 30)	28 (26,31)	29 (26, 32)	29 (26, 31)	<0.001
Education, % (n)*							<0.001
Illiterate	14.1	14.7	14.5	3.7	4.2	4.0	
Primary school	21.4	24.0	23.1	12.2	14.5	13.8	
Junior high school school	52.6	48.9	50.2	58.9	57.4	57.9	
Senior high school school or above	9.9	9.4	9.6	24.0	19.7	21.1	
Did not know	2.0	3.0	2.7	1.2	4.2	3.2	
**Grandparents**							
Age in years (median (Q1, Q3))	50 (46, 55)	52 (48, 58)	51 (47, 57)	52 (49, 57)	54 (51, 59)	54 (50, 59)	<0.001
Education, % (n)							0.057
Illiterate	66.7	69.9	69.1	57.3	61.0	60.3	
Primary school	19.8	17.5	18.1	26.5	21.6	22.6	
Junior high school	12.5	10.3	10.9	16.2	14.9	15.1	
Senior high school	0.5	1.5	1.2	0.0	1.4	1.1	
Did not know	0.5	0.8	0.7	0.0	1.1	0.9	

^a^Baseline vs follow-up for the 6–23 months age group.

#### Caregivers’ infant and young child knowledge and practices

As shown in Table 2, caregivers’ feeding knowledge was still poor despite having improved over the years. Less than 10% of caregivers knew that breastfeeding could be continued up to two years, less than 50% of caregivers knew the accurate duration of exclusive breastfeeding, and only around 20% of caregivers knew to start feeding children with meat from the age of 6–8 months. Similarly, infant feeding practices were suboptimal and mostly did not change, except for children who were given iron-rich or iron-fortified foods, which increased from 41.5% in 2012 to 70.9% in 2018 (P<0.001). Although more caregivers ever received breastfeeding and complementary feeding information during pregnancy or after delivery in 2018 than in 2012 ((breastfeeding information: 26.0% vs 35.2%, P<0.001; complementary feeding information: 16.2% vs 25.9%, P<0.001), the proportion was only around 30%.

**Table 2:** Caregivers’ infant and young child feeding knowledge and practices in both surveys.

Indicators	Survey in 2012			Survey in 2018			*P-*values
	n^a^	N^b^	Percentage (%)	n^a^	N^b^	Percentage (%)	
**Feeding knowledge**							
Caregivers knowing the duration of exclusive breastfeeding	336	1804	18.6	298	754	39.5	<0.001
Caregivers knowing continued breastfeeding until two years	42	1804	2.3	47	754	6.23	<0.001
Caregivers knowing introduction of complementary foods at 6–8 months	779	1804	43.2	485	754	64.3	<0.001
Caregivers knowing starting feeding children with meat at 6–8 months	383	1804	21.2	148	754	19.6	0.362
**Feeding practices**							
Children breastfed until two years^c^	39	502	7.8	15	179	8.4	0.068
Children given complementary foods at 6–8 months ^d^	259	319	81.2	101	123	82.1	0.823
Children aged 6–23 months given iron-rich or iron-fortified foods during the past 24 hours	749	1804	41.5	533	754	70.7	<0.001
Children aged 6–23 months were given meat during the past 24 hours	715	1804	39.6	329	754	43.6	0.061
Minimum dietary diversity	929	1804	51.5	420	754	55.7	0.052
Minimum meal frequency	508	1804	28.2	211	754	28.0	0.928
Minimum acceptable	188	1804	10.4	90	754	11.9	0.262
**Information sources**							
Caregivers ever received breastfeeding information during pregnancy or after delivery	469	1804	26.0	265	754	35.2	<0.001
Caregivers ever received complementary feeding information during pregnancy or after delivery	293	1804	16.2	195	754	25.9	<0.001

^a^Number of caregivers who responded positive on the knowledge/practices.

^b^Total number of mother eligible for the question.

^c^ Only children aged 20 to 23 months were used to calculate this indicator (From “Indicators for assessing infant and young child feeding practices”).

^d^Only children aged 6 to 8 months were used to calculate this indicator (From “Indicators for assessing infant and young child feeding practices”).

**Fig 1:**
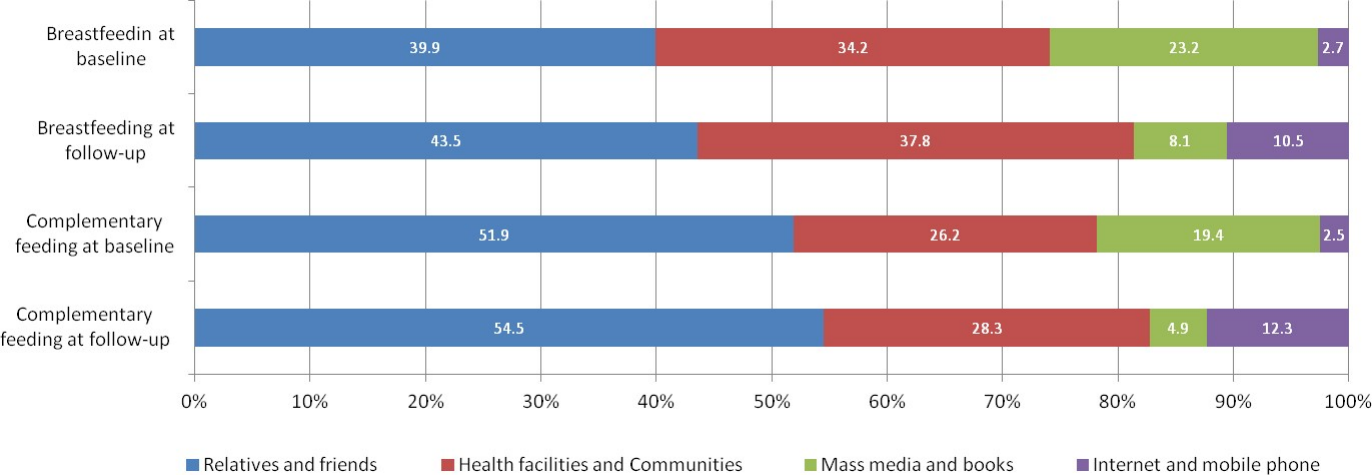
Distribution of sources for infant and young child feeding information.

Sources of infant feeding information. Around 50% of caregivers reported having received feeding information from their relatives and friends, followed by health facilities and communities accounting for about 30% in both surveys (Fig 1). Information from mass media and books dropped from around 20% in 2012 to less than 10% in 2018, whereas Internet and mobile phones as an information source increased to more than 10% in 2018.

#### Use of smartphone and WeChat

Smartphone and WeChat app usage were very popular among mothers in the survey in 2018. More than 80% of mothers were currently using both smartphone and WeChat app in their smartphones (Table 3). However, only around half of the grandparents were currently using smartphones, and less than 30% of them used WeChat app. Nearly 80% of mothers used WeChat for more than one hour every day. Around 30% of caregivers followed Infant and young child feeding official accounts, and 75.4% of them were willing to receive information from WeChat official accounts.

**Table 3:** Mothers’ and grandparents’ use of smartphones and WeChat in the survey in 2018 (N = 493^a^).

	Mothers		Grandparents		Total	
	(n/N)	%	(n/N)	%	(n/N)	%
**Smartphone use**						
Currently using smartphones	(260/292)	89.0	(106/201)	52.7	(366/493)	74.2
Smartphone can access the internet	(236/260)	90.8	(56/106)	52.8	(292/366)	79.8
Ways to access the internet						
WiFi at home or the workplace	(157/258)	60.9	(43/62)	69.4	(200/320)	62.5
Mobile traffic	(96/258)	37.2	(17/62)	27.4	(113/320)	35.3
Other	(5/258)	1.9	(2/62)	3.2	(7/320)	2.2
**WeChat use**						
Currently using WeChat	(236 /292)	80.8	(58/201)	28.9	(294/493)	59.6
Using WeChat for more than one hour every day	(183/236)	77.5	(33/58)	56.9	(286/294)	73.5
Following infant and young child feeding official accounts	(92/236)	39.0	(7/58)	12.1	(99/294)	33.7
Willing to receive information from official accounts	(178/236)	75.4	(34/58)	58.6	(212/294)	72.1

^**a**^ Among 754 interviewed caregivers, there were 292 mothers and 201 **grandparents**.

### Qualitative semi-structured interviews’ results

#### Participants in the qualitative interviews

A total of 17 pregnant women in their second or third trimester and 16 mothers who had a child aged 0–6 months participated in the semi-structured interviews in September 2018. The median age was 29 (ranged from 18 to 45) for pregnant women and 31 (ranged from 23 to 40) for mothers. The median gestational weeks for pregnant women were 29 weeks, ranged from 14 to 38 weeks. We interviewed seven mothers who had a children less than one week, one mother who had a child aged 2 months, and eight mothers who had children aged 4–5 months. Most of pregnant women and mothers attended junior high school or above, with four of them only attending primary school and two being illiterate. Five pregnant women and two mothers had a job, such as being a teacher, technician, or commercial service worker, and the others stayed at home for housework.

#### Mothers’ breastfeeding knowledge and practices in qualitative interviews

In the semi-structured interviews, pregnant women and mothers generally did not have a clear idea about exclusive breastfeeding and its recommended duration. All the interviewed pregnant women thought children should be given water during the exclusive breastfeeding period (up to six months after delivery) due to the following reasons: children may get sweaty, be thirsty, their mouths may be dry, water can replenish children’s energy and because other peoples’ advice.

Interviewed pregnant woman 1 (aged 37 years, 37 weeks of gestation, primary school education, shuangshu township) said, “Children may be thirsty (if fed only by breast milk), just like adults drink milk, milk is milk, water is water.”

Mothers’ reasons to give water to their children were: children were thirsty, refused breast milk, their stools were dry, no breast milk after delivery, hospital information. In addition, mothers fed formula or noodles to their children younger than six months, as they thought breast milk was not enough for children. The duration of exclusive breastfeeding given by pregnant women and mothers varied from 3 months to 12 months.

Interviewed mother 1 (aged 28 years, child aged 5 months, junior college education, Xishan Township) said, “Sometimes, my child’s digestion was not good, that is, his stool was dry, I would give him some water to help him digest.”

Interviewed mother 2 (aged 31 years, child aged 2 months, middle school education, Shuangshu Township) said, “The doctor told me I should give some water to my child.”

#### Sources of infant feeding information in qualitative interviews

In the semi-structured interviews, pregnant women and mothers said that they received feeding information from the Internet (Baidu search engine) (n = 18), hospitals (n = 17), apps on their smartphones (n = 13), the elder people (n = 7), their own experience (n = 4), maternal and child health booklets developed by local health institutions (n = 2), and books (n = 1).

Although the Internet seemed to be one of the most important sources for feeding information, pregnant women and mothers did not completely believe this information. They often referred to comments from other people, health workers, elder people at home or books before they accepted a specific piece of information.

*Interviewed pregnant woman 2 (aged 24 years, 23 weeks of gestation, middle school education, Weiyuan Township) said:” I still believe doctors, and the information from the internet is only as a reference”*.

Interviewed mother 3 (aged 27 years, child aged 5 days, junior college education, Weiyuan township)said: “I often searched several browsers at the same time and reviewed comments from other people, but I did not believe completely most of the time.”

*Interviewed mother 4 (aged 24 years*, *child aged 1 days*, *technical secondary school* *education*, *Weiyuan Township) said*: *“I often read the information which had the most comments*.*”*

#### WeChat usage of mothers in qualitative interviews

In the semi-structured interviews, all the pregnant women and mothers had smartphones and used WeChat, and most of them used WeChat at least more than one year. Messaging, moments, official accounts, news, and WeChat group were the most used functions. However, only one pregnant woman and two mothers followed the infant and young child feeding-related official accounts, and they explained that they had never heard of those official accounts (n = 20), or they thought it was unnecessary (n = 3). Participants who followed could read and review the contents in official accounts when they were free.

Interviewed pregnant woman 3 (aged 27 years, 14 weeks of gestation, middle school education, Shuangshu Township) said: “My (previous) children are very healthy, so I did not follow (the infant and young child feeding official accounts).”

#### Use experience of the WeChat feeding health education platform in qualitative interviews

Most interviewed pregnant women and mothers stated that the interface of the module was good, and they liked it. One mother said using the interface was not so smooth.

For the *feeding messages* module, four pregnant women and five mothers liked pictures and text for feeding knowledge as they thought it was easy to understand, they got used to reading text, or they thought videos were too fast or a bit slow. Seven pregnant women and eight mothers liked videos for feeding knowledge as they believed that videos were easy to understand, simple and convenient to watch. Other pregnant women and mothers expressed that pictures, text and videos were ‘OK’ for them. Most pregnant women and mothers said that they were interested in the feeding lecture classroom, and that the contents were easy to understand and useful.

Pregnant women wanted to know information on breastfeeding positions, how to increase breast milk supply, spitting up, child health, how to hold a baby, cough, sleep and antenatal care. Mothers wanted to know more about breastfeeding and complementary feeding, food allergies, illnesses, and growth development.

For the *Baby Growth Chart* module, five pregnant women and three mothers expressed that they could not understand the growth chart after they filled in the growth data.

Thirteen pregnant women and 14 mothers were willing to use this function to monitor the growth of their children. However, *interviewed* mother 5 *(aged 33 years*, *child aged 2 days*, *Primary school* *education*, *Weiyuan Township)* said: *“I don’t understand the function*, *so I would not use it*.*”*

## Discussion

### Main findings

The results of this study indicate that both pregnant women and caregivers had limited knowledge about exclusive breastfeeding and complementary feeding in our research setting in rural China. The key IYCF indicators were suboptimal and did not improve over the years. Furthermore, there was an absence of accurate information sources on infant feeding and child nutrition, and caregivers mostly received feeding information from their relatives and friends. Smartphones and WeChat app were widely used among pregnant women and mothers in this setting.

### New channels are needed to deliver accurate infant feeding recommendations

Improved feeding knowledge and attitudes toward breastfeeding and complementary feeding are related to more positive child health outcomes [39, 40]. Our study indicated that the traditional infant feeding counseling provided by rural health facilities were of low quality and most of the feeding practices did not significantly improve over time, and around two-thirds of caregivers received no advice on infant feeding from health facilities. Relatives and friends were the main source of caregivers’ feeding knowledge, however, they are unlikely to have access to better information and may have misinformed mothers [17–19]. Therefore, new channels are needed in rural China to deliver accurate infant feeding information and to improve child health.

### WeChat could be a potential way to deliver infant feeding recommendations

In our study, nearly 80% of mothers in the study areas used WeChat, and around 70% used WeChat more than one hour every day. Women could easily access to the internet on their smartphones, as WiFi was available at home or at the workplace. Although only 39% of mothers in the study areas followed child feeding official accounts, more than three-fourths were willing to receive feeding information in the format of text, pictures or videos through WeChat. The WeChat feeding health education platform developed by our study team was generally accepted by women, as the information was easy to understand and useful. Our platform was based on the WeChat official account of the local Maternal and Child Health Family Planning Service Centre which was trusted by women. Studies have shown that WeChat official accounts have potentials to positively impact health behavior [19–22]. Future studies are needed to test the effectiveness of a WeChat-based health education platform on key infant feeding practices in rural China.

Our study has some limitations. This study took place in one Chinese rural county and caution is needed when generalizing the findings from this study to other settings. In addition, we did not conduct qualitative interview among mothers who had children aged 6–23 months, which may not get their depth opinions on WeChat as intervention to deliver infant and young child feeding information. However, data from the survey in 2018 indicated that 75.4% of mothers aged 6–23 months were willing to received feeding information from WeChat official accounts.

## Conclusions

Our study indicates that caregivers’ feeding knowledge and practices were poor in Huzhu County, and there was an absence of accurate information sources on infant feeding and child nutrition. The WeChat-based feeding health education platform was generally accepted by mothers and could also be explored in future studies to be a new channel for delivery of IYCF recommendations.

## Supporting information

S1 Data Data for two cross-sectional surveys. (XLSX)

S1 File Read me (Explanation of all the variables in the database). (XLSX)

S2 File Questionnaire for the cross-sectional surveys. (DOCX)
