# Supplementary material for: Feasibility of using WeChat to improve infant and young child feeding in rural areas in China: A mixed quantitative and qualitative study
Source: PLoS One. 2021 Feb 25;16(2):e0246942. doi: 10.1371/journal.pone.0246942 (PMC7906387; doi:10.1371/journal.pone.0246942)
Supplement: S2 File — (DOCX) [file pone.0246942.s003.docx]

1. **Family number information（ID）**

| **ID.1a** | County：___________ | **ID.1an**□□ |
| --- | --- | --- |
| **ID.1b** | Township：___________ | **ID.1bn**□□ |
| **ID.1c** | Village：___________ | **ID.1cn**□□ |
| **ID.2** | Name of interviewee：___________ | **ID.2a**□□ |
| **ID.3** | Relationship of interviewee and child： | **ID.3**□ |
|  | 1.mother 2.father 3.grandparents 9．Others |  |
| **ID.4** | Are you the main caregiver of the child? 1.yes 2.no | **ID.4**□ |
| **ID.5** | Name of the youngest child from 6 to 23 months of age:_________________ | **ID.5** |
| **ID.6** | Child sex：1.male 2.female | **ID.6**□ |
| **ID.7** | Child birthdate（Solar calendar）：  __ __ __ __y__ __m__ __d | **ID.7** □□□□/□□/□□ |
| **ID.8** | Birthweight：__ __ __ __ g（*Don’t know =8888*） | **ID.8**□□□□ |
| **ID.9** | Birthlength：__ ____cm（*Don’t know =88.8*） | **ID.9**□□.□ |
| **ID.10** | gravidity  __ times（*if≥7，fill“7”，8.Don’t know*） | **ID.10**□ |
| **ID.10a** | parity __ （*if≥7，fill“7”，8. Don’t know*） | **ID.10a**□ |
| **ID.11** | Was the mother anemic during pregency？ | **ID.11**□ |
|  | 1.Yes 2.No 8. Don‘t know |  |
| **ID.12** | How long has the baby been pregnant? (example: 38 weeks and 5 days are recorded as 38 weeks) __ __weeks（88. Don‘t know） | **ID.12**□□ |
| **ID.13** | Hukou？1.Urban 2.Rural | **ID.13**□ |
| **ID.14** | Interviewer：___________ | **ID.14**□□ |
| **ID.15** | Survey date：__ __ __ __y__ __m__ __d | **ID.15**□□□□/□□/□□ |

1. **Breast-feeding and nutrition（BN）**

| **I'd like to ask how children should be fed,** **these questions do not refer specifically to your children, but generally to children under the age of two.** | | |
| --- | --- | --- |
| **BN.1** | Can you tell me until what age a baby should only receive breastmilk, i.e. no other food, water or fourmal) | **BN.1**□□ |
|  | __ __months [0=less than one month, 88=Don’t know] |  |
| **BN.2** | Can you tell me until what age a baby should start receiving foods such as mashed or solid foods? | **BN.2**□□ |
|  | __ __ months [0=less than one month, 88=Don’t know] |  |
| **BN.3** | Can you tell me until what age a baby should be breastfed? | **BN.3**□□ |
|  | __ __months [0=less than one month, 88=Don’t know, 90=when the milk dries out or when the child no longer wants the breast] |  |
| **BN.4.f** | Can you tell me until what age a baby should be given meat? | **BN.4.f**□□ |
|  | __ __ months [0=less than one month, 88=Don’t know] |  |

| **Now let me ask you how you feed your kids.** | | |
| --- | --- | --- |
| **BN.5** | Has your child ever been breastfeed? | **BN.5**□ |
|  | 1. Yes 2. No **——>**  ***ship to BN.6a*** |  |
| **BN.6** | Was your child breastfed yesterday during the day or at night？ | **BN.6**□ |
|  | 1. Yes——>How many times did you breastfeed your child during the past 24 hours? __ __ *times [****填在BN.6.a中，****88=Don’t know]***——>**  ***ship to BN.7***  2. No.The baby hasn't been weaned, but it didn't eat yesterday.**——>**  ***ship to BN.7*** | **BN.6.1**□□ |
|  | 3. No. My child has been stopped breastfeeding**——>** When did you completely stop breastfeeding to your child?__ __ months*[****fill in BN.6.b，****88=Don’t know ]* | **BN.6.3**□□ |
|  | 8.  *Don’t know* **——>**  ***ship to BN.7*** |  |
| **BN.6a** | Why didn't you breast feed your baby or why did you wean your child? | **BN.6a**□ |
|  | 1. No milk / no milk  2. Breast milk is not nutritious  3. The child is old  4. Mom goes to work  5. Mother is sick  6. Other details_______________________________________  8. I don't know |  |

| **BN.7** | I want to know about the vitamins, medicine and liquids that the child took yesterday( Including day and night.) What did the child eat?  **[** **Read item by item, record item by item ]** | | |
| --- | --- | --- | --- |
| **Vitamin 、 medicine or liquids** | | **Have your kids eaten in the last 24 hours?** |  |
| 1. Vitamin, mineral supplements or medicine? | | 1. Yes 2. No 8. Don’t know | BN.7.1□ |
| 1. ORS | | 1. Yes 2. No 8. Don’t know | BN.7.2□ |
| 1. Plain water? | | 1. Yes 2. No 8. Don’t know | BN.7.3□ |
| 1. Infant formula | | 1. Yes 次*(****fill BN7.4.1，****88. Don’t know)*  2. No 8. Don’t know | BN.7.4□  BN.7.4.1□□ |
| 1. Milk | | 1. Yes 次*（* ***fill BN.7.5.1，****88. Don’t know）*  2. No 8. Don’t know | BN.7.5□  BN.7.5.1□□ |
| 1. Juice or juice drinks? | | 1. Yes 2. No 8. Don’t know | BN.7.6□ |
| 1. clear broth? | | 1. Yes 2. No 8. Don’t know | BN.7.7□ |
| 1. yogurt? | | 1. Yes 次*（* ***fill BN.7.8.1，****88. Don’t know）*  2. No 8. Don’t know | BN.7.8□  BN.7.8.1□□ |
| 1. thin porridge? | | 1. Yes 2. No 8. Don’t know | BN.7.9□ |
| 1. lactic acid beverage | | 1. Yes 2. No 8. Don’t know | BN.7.10□ |
| 1. other liquids:___________ | | 1. Yes 2. No 8. Don’t know | BN.7.11□ |

| **BN.8** | I want to know all the food the child ate yesterday, including what he ate at home and outside | | | | |
| --- | --- | --- | --- | --- | --- |
| **Foods** | | | **Have your kids eaten in the last 24 hours?** |  | |
| 1. Porridge, bread, rice, noodles, or other foods made from grains | | | 1. Yes 2. No 8. Don’t know | BN.8.1□ | |
| 1. Pumpkin, carrots, squash, or sweet potatoes that are yellow or orange inside | | | 1. Yes 2. No 8. Don’t know | BN.8.2□ | |
| 1. White potatoes, white yams, manioc, cassava, or any other foods made from roots | | | 1. Yes 2. No 8. Don’t know | BN.8.3□ | |
| 1. any dark green leafy vegetables | | | 1. Yes 2. No 8. Don’t know | BN.8.4□ | |
| 1. ripe mangoes, ripe papayas, or (insert other local vitamin a-rich fruits) | | | 1. Yes 2. No 8. Don’t know | BN.8.5□ | |
| 1. any other fruits or vegetables | | | 1. Yes 2. No 8. Don’t know | BN.8.6□ | |
| 1. liver, kidney, heart, or other organ meats | | | 1. Yes 2. No 8. Don’t know | BN.8.7□ | |
| 1. any meat, such as beef, pork, lamb, goat, chicken, or duck | | | 1. Yes 2. No 8. Don’t know | BN.8.8□ | |
| 1. any fat | | | 1. Yes 2. No 8. Don’t know | BN.8.9□ | |
| 1. eggs | | | 1. Yes 2. No 8. Don’t know | BN.8.10□ | |
| 1. fresh or dried fsh, shellfsh, or seafood | | | 1. Yes 2. No 8. Don’t know | BN.8.11□ | |
| 1. any foods made from beans, peas, lentils, nuts, or seeds | | | 1. Yes 2. No 8. Don’t know | BN.8.12□ | |
| 1. cheese, yogurt, or other milk products | | | 1. Yes 2. No 8. Don’t know | BN.8.13□ | |
| 1. any oil, fats, or butter, or foods made with any of these | | | 1. Yes 2. No 8. Don’t know | BN.8.14□ | |
| 1. any sugary foods such as chocolates, sweets, candies, pastries, cakes, or biscuits | | | 1. Yes 2. No 8. Don’t know | BN.8.15□ | |
| 1. condiments for favor | | | 1. Yes 2. No 8. Don’t know | BN.8.16□ | |
| 1. ham sausage | | | 1. Yes 2. No 8. Don’t know | BN.8.17□ | |
| **Record the other foods mentioned by the mother that are not listed in the table above：** | | | | | |
| **BN.9** | | Has your child ever eaten solid, semi-solid or paste food? When did he eat for the first time? | | | **BN.9**□□ |
|  |  | 1. Yes, for the first time at the age of __ __ months (fill in 00 for less than 1 month) 2. No solid or semi-solid food has been fed to the child yet**——>** ***ship to* BN.12a** | | | **BN.9.1**□□ |
| **BN.10** | | how many times did (NAME) eat solid, semi-solid, or soft foods other than liquids yesterday during the day or at night? | | | **BN.10**□ |
|  |  | ___ times *[ if≥7，fill“7”；8. Don’t know]* | | |  |
| **BN.11** | | Do you usually cook for children alone? | | | **BN.11**□ |
|  |  | 1. Cook alone most of the time  2. Don't cook for children alone, but do it with adults  8. Don’t know | | |  |
| **Now I'd like to ask about the extra iron supplements or supplements that kids eat. Tell me if the child ate the food, and if it's eaten with other foods, it's also eaten.** | | | | | |
| **BN.12.a** | | Yesterday, during the day or night, did your child consume any food to which you added a powder or sprinkles contained iron?  **[** **Display common iron fortified powders or sprays or tablets.]** | | | **BN.12.a**□ |
|  |  | 1. Yes 2. No 8. Don’t know | | |  |
| **BN.12.b** | | Yesterday, during the day or night, did your child consume any lipid contianed iron?  **[ Show mother the common local liquid nutrient supplements.]** | | | **BN.12.b**□ |
|  |  | 1. Yes 2. No 8. Don’t know | | |  |

| **I'd like to ask if you've received information or guidance on breastfeeding.** | | |
| --- | --- | --- |
| **BN.13** | Have you ever received breastfeeding information before and after delivery? | **BN.13**□ |
|  | 1. Yes  2. No **——>** **ship to BN.14**  8. Don’t know **——>** **ship to BN.14** |  |

| **BN.13.a** | Where do you get information about breastfeeding?  **[** **Don't prompt, record all access to information.]** | | |
| --- | --- | --- | --- |
|  | **1. Family and friends**  11 Family members  12 Friends or neighbors | 1. Yes 2. No  1. Yes 2. No | **BN.13.a.11**□  **BN.13.a.12**□ |
|  | **2. Public medical institutions**   \| 21 Hospital of county or above \| \| --- \| \| 22 MCH hospital \| \| 23 CHC \| \| 24 Township hospital \| \| 25 CHS \| \| 26 Village clinic \| | 1. Yes 2. No  1. Yes 2. No  1. Yes 2. No  1. Yes 2. No  1. Yes 2. No  1. Yes 2. No | **BN.13.a.21**□  **BN.13.a.22**□  **BN.13.a.23**□  **BN.13.a.24**□  **BN.13.a.25**□  **BN.13.a.26**□ |
|  | **3. Private medical institutions**   \| 31 Private Hospital \| \| --- \| \| 32 Private clinic \| \| 33 Pharmacy \| | 1. Yes 2. No  1. Yes 2. No  1. Yes 2. No | **BN.13.a.31**□  **BN.13.a.32**□  **BN.13.a.33**□ |
|  | **4. community**   \| 41 Brith attendant \| \| --- \| \| 42 Family planing staff \| | 1. Yes 2. No  1. Yes 2. No | **BN.13.a.41**□  **BN.13.a.42**□ |
|  | **5. mass media**   \| 51 Internet \| \| --- \| \| 52 newspaper /magazine \| \| 53 Radio/TV \| \| 54 Mobile Message \| | 1. Yes 2. No  1. Yes 2. No  1. Yes 2. No  1. Yes 2. No | **BN.13.a.51**□  **BN.13.a.52**□  **BN.13.a.53**□  **BN.13.a.54**□ |
|  | 1. **61. books** | 1. Yes 2. No | **BN.13.a.61□** |
|  | 1. **71.Other,**detailed description_**________________________** | 1. Yes 2. No | **BN.13.a.71□** |

| **I would like to ask if you have received any information or guidance on complementary food supplements.** | | | |
| --- | --- | --- | --- |
| **BN.15** | Have you ever received compelmentarry feeding information before and after delivery? | | **BN.15**□ |
|  | 1. Yes  2. No **——>**  ***End the BN section and move on to the next section***  8. Don’t know **——>**  ***End the BN section and move on to the next section*** | |  |
| **BN.15.a** | Where do you get advice on adding complementary food to your child?  **[** **Record only, don't read options or tips. Record all channels to get advice.]** | |  |
|  | **1. Family and friends**  11 Family members  12 Friends or neighbors | 1. Yes 2. No  1. Yes 2. No | **BN.15.a.11**□  **BN.15.a.12**□ |
|  | **2. Public medical institutions**   \| 21 Hospital of county or above \| \| --- \| \| 22 MCH hospital \| \| 23 CHC \| \| 24 Township hospital \| \| 25 CHS \| \| 26 Village clinic \| | 1. Yes 2. No  1. Yes 2. No  1. Yes 2. No  1. Yes 2. No  1. Yes 2. No  1. Yes 2. No | **BN.15.a.21**□  **BN.15.a.22**□  **BN.15.a.23**□  **BN.15.a.24**□  **BN.15.a.25**□  **BN.15.a.26**□ |
|  | **3. Private medical institutions**   \| 31 Private Hospital \| \| --- \| \| 32 Private clinic \| \| 33 Pharmacy \| | 1. Yes 2. No  1. Yes 2. No  1. Yes 2. No | **BN.15.a.31**□  **BN.15.a.32**□  **BN.15.a.33**□ |
|  | **4. community**   \| 41 Brith attendant \| \| --- \| \| 42 Family planing staff \| | 1. Yes 2. No  1. Yes 2. No | **BN.15.a.41**□  **BN.15.a.42**□ |
|  | **5. mass media**   \| 51 Internet \| \| --- \| \| 52 newspaper /magazine \| \| 53 Radio/TV \| \| 54 Mobile Message \| | 1. Yes 2. No  1. Yes 2. No  1. Yes 2. No  1. Yes 2. No | **BN.15.a.51**□  **BN.15.a.52**□  **BN.15.a.53**□  **BN.15.a.54**□ |
|  | 1. **61. books** | 1. Yes 2. No | **BN.15.a.61**□ |
|  | 1. **71.Other,**detailed description_**_______________________** | 1. Yes 2. No | **BN.13.a.71□** |

### 6. Basic family information

| **HH.1** | Household size（Record the number of family members） __ __ | | **HH.1**□□ |
| --- | --- | --- | --- |
| **HH.4** | Age of monther：__ __years old | | **HH.4**□□ |
| **HH.5** | Ethnic of mother | | **HH.5**□ |
|  | 1. Han nationality   2. Hui nationality   1. Tu nationality 2. Tibetan nationality | 1. Salar nationality 2. Mongolian nationality 3. Others_____________ |  |
| **HH.6** | Education of mother | | **HH.6**□ |
|  | 1. Never went to school  2. Primary school  3. Junior high school  4. High school / technical school | 5. Technical secondary school / technical secondary school  6. Junior College  7. University or above  8. Don‘t know |  |
| **HH.7** | How many years did mother go to school? | | **HH.7**□□ |
|  | __ __ years （00. Never went to school，88. Don’t know） | |  |
| **HH.8** | Mother's work | | **HH.8**□□ |
|  | 1. Housework  2. Persons in charge of government organs, enterprises and institutions  3. Professional and technical personnel  4. Business service personnel  5. Production personnel of agriculture, forestry, animal husbandry, fishery and water conservancy | 6. Operators of production and transportation equipment  7. Soldiers  8. Mining  9. Architecture  10. Others__________________ |  |
| **HH.9** | Did mother work outside during last 12 months？ | | **HH.9**□ |
|  | 1.Yes  2.No***——>ship to HH.10a*** | |  |
| **HH.9a** | How old was the child when the mother began to work? __ __months**（Fill in 00 for less than one month, Don’t know fill in 88）** | | **HH.9a**□□ |
| **HH.9b** | Accumulated working time of mother：__ __ months**（From August last year to now, Fill in 00 for less than one month, Don’t know fill in 88）** | | **HH.9b**□□ |
| **HH.9c** | How often does mother go home in the past year?  1. < 3 months 2.3-5 months 3.6-11 months 4. > = 12 months 5. Don’t know | | **HH.9c**□ |
| **HH.10** | Age of father: __ __years old | | **HH.10**□□ |
| **HH.11** | Ethnic of father | | **HH.11**□ |
|  | 1.Han nationality  2.Hui nationality  3.Tu nationality  4.Tibetan nationality | 5.Salar nationality  6.Mongolian nationality  7.Others_____________ |  |
| **HH.12** | Education of father | | **HH.12**□ |
|  | 1. Never went to school  2. Primary school  3. Junior high school  4. High school / technical school | 5. Technical secondary school / technical secondary school  6. Junior College  7. University or above  8. Don‘t know |  |
| **HH.12a** | How many years did father go to school? | | **HH.12a**□□ |
|  | __ __ years （00. Never went to school，88. Don’t know） | |  |
| **HH.13** | Father's work | | **HH.13**□□ |
|  | 01. Housework  02. Persons in charge of government organs, enterprises and institutions  03. Professional and technical personnel  04. Business service personnel  05. Production personnel of agriculture, forestry, animal husbandry, fishery and water conservancy | 06. Operators of production and transportation equipment  07. Soldiers  08. Mining  09. Architecture  10. Others__________________ |  |
| **HH.14** | Did father work outside during last 12 months? | | **HH.14**□ |
|  | 1. Yes   1. 2. No***——>ship to HH.16*** | |  |
| **HH.14a** | How old was the child when the father began to work? __ __months**（Fill in 00 for less than one month, Don’t know fill in 88）** | | **HH.14a**□□ |
| **HH.14b** | Accumulated working time of father：__ __ months**（From August last year to now, Fill in 00 for less than one month, Don’t know fill in 88）** | | **HH.14b**□□ |
| **HH.14c** | How often does mother go home in the past year?  1. < 3 months 2.3-5 months 3.6-11 months 4. > = 12 months 5. Don’t know | | **HH.14c**□ |
| **HH.16** | Who are the main caregivers of children? | | **HH.16**□ |
|  | 1. mother***——>ship to HH.17*** 2. father***——>ship to HH.17*** 3. grandparents 4. ohers ______________________ | |  |
| **HH.16a** | Age of primary caregiver other than parents? __ __ years old | | **HH.16a**□□ |
| **HH.16b** | Education level of the main caregiver other than parents | | **HH.16b**□ |
|  | 1. Never went to school  2. Primary school  3. Junior high school  4. High school / technical school | 5. Technical secondary school / technical secondary school  6. Junior College  7. University or above  8. Don‘t know |  |
| **HH.16c** | How many years of schooling have the main caregivers other than parents attended? | | **HH.16c**□□ |
|  | __ __ years（00.Never went to school，88. Don’t know） | |  |

| **HH.16d** | A major parenting profession other than parents | | **HH.16d**□□ |
| --- | --- | --- | --- |
|  | 1. Housework  2. Persons in charge of government organs, enterprises and institutions  3. Professional and technical personnel  4. Business service personnel  5. Production personnel of agriculture, forestry, animal husbandry, fishery and water conservancy | 6. Operators of production and transportation equipment  7. Soldiers  8. Mining  9. Architecture  10. Others__________________ |  |
| **HH.17** | What is the main source of your family's income? | | **HH.17**□ |
|  | 1. Planting food  2. Planting vegetables  3. Animal husbandry  4. Forestry | 5. Doing business  6. Working  7. Others___________  8. I don't know |  |

7. Mobile phone usage (MP) (Only for survey in 2018)

| **Now I'd like to know about the use of your mobile phone.** | |
| --- | --- |
| **MP.1**  Do you use your mobile phone? | **MP.1**□ |
| 1. yes 2. no——> ***End the section*** |  |
| **MP.2**  What brand and model is the mobile phone in use now? |  |
| brand and model :____________（8= Don‘t know ） |  |
| **MP.3**  Is your mobile phone a smart phone?**（** **The investigator looks at the phone and checks the operating system before filling in.）** | **MP.3**□ |
| 1. yes 2. no ——>**ship to MP.5**   8. Don‘t know ——>**ship to MP.5** |  |
| **MP.4**  Do you use your mobile phone to access the Internet?   1. yes 2. no ——> ***End the section***   8. Don‘t know ——> ***End the section*** | **MP.4**□ |
| **MP.4a**  How do you charge for Internet access by mobile phone? (multiple choice) | **MP.4a**□ |
| 1. Charging by traffic ； 2. Using traffic package ——>MP10.a □□□M/ Monthly Internet traffic 3. Use free WiFi at home or at work 4. Other details :_______________   8. Don‘t know |  |
| **MP.5** Do you usually use wechat?  1. yes  2. no ——> ***End the section*** | **MP.5**□ |
| **MP.8** How long do you use wechat every day?  1.Less than 1 hour  2.1-2 hours  3.2-3 hours  4.More than 3 hours  5.More than 4 hours | **MP.8**□ |
| **MP.12** Do you pay attention to the subscription number or service number of infant feeding (breastfeeding and supplementary food supplement) on wechat?  1. yes  2. no | **MP.12**□ |
| **MP.1**2a Do you want to get the knowledge and information about infant feeding (breastfeeding and complementary feeding) through wechat？  1. yes  2. no | **MP.12a**□ |
